# Atrial fibrillation detection performance of an insertable cardiac monitor: Results from an Assert-IQ post-market clinical study and a novel artificial intelligence algorithm

**DOI:** 10.1016/j.hroo.2025.10.021

**Published:** 2025-11-17

**Authors:** Ulrika Birgersdotter-Green, Willibaldo Ojeda, Harish Manyam, Alvaro Manrique Garcia, George E. Manoukian, Mohammad-Ali Jazayeri, Frank Cuoco, Frederick Han, Michael Katcher, Rakesh Gopinathannair, Dale Yoo, Lin Feng, Fujian Qu, Wenjiao Lin, Kwangdeok Lee, Vishnu Charan, Suneet Mittal, Dhanunjaya Lakkireddy

**Affiliations:** 1 University of California at San Diego Medical Center, San Diego, California; 2 Shannon Clinic, San Angelo, Texas; 3 Erlanger Medical Center, Chattanooga, Tennessee; 4 Heart Rhythm Associates, Shenandoah, Texas; 5 Trinity Health-Michigan d/b/a Michigan Heart, Ann Arbor, Michigan; 6 Trident Medical Center, Charleston, South Carolina; 7 Mass General Brigham-Salem Hospital, Salem, Massachusetts; 8 Kansas City Heart Rhythm Institute, Overland Park, Kansas; 9 Heart Rhythm Specialists, McKinney, Texas; 10 Abbott, Sylmar, California; 11 Valley Hospital and the Snyder Center for Comprehensive Atrial Fibrillation, Paramus, New Jersey

**Keywords:** Atrial fibrillation, Insertable cardiac monitor, Assert-IQ, Artificial intelligence, AF detection algorithm, Holter monitoring, False-positive reduction, Rhythm monitoring, AF burden quantification

## Abstract

**Background:**

Accurate atrial fibrillation (AF) detection and burden assessment are critical features of modern insertable cardiac monitors (ICMs), enabling precise determination of AF episode patterns, frequency, duration, and total burden to guide treatments.

**Objective:**

This study aimed to evaluate the AF detection performance of the Assert-IQ ICM and assess the impact of an artificial intelligence (AI) algorithm designed for reducing false-positive AF episodes.

**Methods:**

This prospective, single-arm, multicenter study enrolled 151 subjects with symptomatic, drug-refractory paroxysmal or persistent AF. A Holter assessment was conducted after ICM insertion. AF detection metrics—sensitivity, specificity, positive predictive value (PPV), and negative predictive value—were evaluated by comparing ICM detections with core laboratory–annotated Holter AF events. The impact of an AI algorithm on AF detection performance was then assessed.

**Results:**

Among 135 analyzable patients, 39 had Holter-confirmed AF with 522 episodes lasting ≥2 minutes. Assert-IQ ICM correctly identified all patients with true AF. Duration-based sensitivity, specificity, PPV, negative predictive value, and accuracy were 93.0%, 99.3%, 97.4%, 98.0%, and 97.9%, respectively. Episode detection sensitivity was 99.4% (gross) and 99.9% (patient average). AF burden correlation between ICM and Holter was excellent (r = 0.99). The AI algorithm retained all true positives and reduced 72.6% of false positives, improving PPV from 79.9% to 93.6%.

**Conclusion:**

Assert-IQ ICM accurately detects AF and quantifies burden for long-term monitoring. The AI algorithm effectively reduces false positives while maintaining high sensitivity.

**Key Findings**

▪ The Assert-IQ insertable cardiac monitor (ICM) demonstrated high sensitivity in detecting atrial fibrillation (AF) and provided accurate quantification of AF burden.
▪ The artificial intelligence (AI) algorithm substantially reduced false-positive AF episodes from the ICM device while fully preserving sensitivity.
▪ The AI algorithm notably improved positive predictive value, particularly for device-detected AF episodes of shorter duration.

## Introduction

Atrial fibrillation (AF) is the most common type of cardiac arrhythmia, affecting 0.4%–1% of the general population, with prevalence increasing with age from less than 1% in young adults to 8% in individuals older than 80 years.^1^, ^2^, ^3^, ^4^ Continuous cardiac monitoring plays a crucial role in managing patients with AF. Insertable cardiac monitors (ICMs) offer advantages over conventional external monitoring methods, including extended monitoring duration, improved patient compliance, and the ability to detect asymptomatic episodes. These capabilities help guide therapeutic decisions regarding antiarrhythmic and anticoagulation therapy and can assess rhythm status in symptomatic and asymptomatic patients.^5^^,^^6^

The performance of AF detection in ICMs made by Abbott has been evaluated across several studies. The DETECT-AF study assessed the performance of Confirm DM2102 ICM and demonstrated high sensitivity for AF detection, establishing a performance baseline for the original Confirm Rx ICM systems.^7^ Subsequent devices, including Confirm Rx and Jot Dx ICM, incorporated algorithm enhancements such as SharpSense technology, which was designed to reduce false positives and improve clinical efficiency.^8^ However, the performance improvements were primarily assessed through retrospective analyses and comparisons with earlier models.^9^ Further evaluation is needed to understand the absolute detection accuracy of Abbott’s latest ICM platform—Assert-IQ ICM.

Meanwhile, cloud-based artificial intelligence (AI) integration has emerged as a promising approach to further reduce the unnecessary data review burden introduced by ICMs.^10^, ^11^, ^12^, ^13^ These AI algorithms are more sophisticated than traditional algorithms incorporated in device firmware and have demonstrated excellent performance in reducing device-detected false positives with minimal loss in sensitivity for certain ICM models. The clinical validation of AI algorithms using AF episodes detected by Assert-IQ ICM and its impact on AF detection performance compared with the gold standard of Holter monitoring has not been done before.

This study was designed to assess the absolute performance of the Assert-IQ ICM’s AF detection algorithm in clinical practice through direct comparison with expert-annotated Holter recordings and to evaluate the impact of an AI-based classification algorithm on the performance of Assert-IQ AF detection.

## Methods

### Study design and participants

The Assert-IQ post-market study (ClinicalTrials.gov identifier: NCT06172699) was a prospective, single-arm, nonrandomized, multicenter study aimed at collecting and evaluating data regarding the performance of the enhanced AF detection algorithm in the Assert-IQ ICM. The study was conducted in accordance with the principles of the Declaration of Helsinki and applicable regulatory requirements. Each institutional review board of the participating sites approved the study, and all patients provided a written informed consent prior to enrollment. All patient data were deidentified prior to analysis to ensure confidentiality. Eligible participants had a clinically approved indication for ICM implantation, such as symptomatic, drug-refractory AF requiring continuous rhythm monitoring, in accordance with current clinical guidelines and physician judgment. They either were scheduled for first-time AF ablation or had ≥2 AF episodes within 1 month prior to enrollment if already implanted with Assert-IQ ICM. Participants needed to be ≥18 years, diagnosed as having drug-refractory AF (unable to tolerate antiarrhythmic drugs [AADs], unwilling to take AADs, or ineffective response to AADs), and able to use the myMerlin application. Exclusion criteria included participation in conflicting studies, existing cardiac devices with pacing capability, a life expectancy of <1 year, or previous AF ablation.

Subjects enrolled in the study were followed for 12 months with scheduled visits at 1 month and 12 months. A Holter assessment was performed at the 1-month visit. Study participants underwent external Holter electrocardiogram (ECG) recording for a minimum of 3 days and up to 7 days. Patients maintained their normal activities and documented any symptoms during the monitoring period. ECG signals were collected using the M5 Recorder (Global Instrumentation) with the 3-lead patient cable. The ICMs were programmed to detect AF episodes lasting ≥2 minutes under the balanced AF sensitivity setting. R-wave sensing maximum sensitivity of 0.125 mV (nominal value) was used if the device-measured R-wave amplitude was ≥0.4 mV. Otherwise, the maximum sensitivity was adjusted to a lower value to ensure ∼3× safety margin for adequate R-wave sensing. The device-stored AF electrogram (EGM) pretrigger duration was set to 30 seconds, and posttrigger duration was set to 60 seconds.

### Holter annotation and episode alignment

An independent core laboratory (IQVIA) analyzed all Holter recordings and annotated the start and end time of each arrhythmic event or period of uninterpretable ECG owing to noise or lead disconnection. The core laboratory was blinded to the device episode detection information. The timestamps of ICM-detected AF episodes were adjusted to match the timestamps used in the Holter recordings to precisely evaluate AF detection performance. In each patient, an ICM-detected AF episode was manually synchronized beat by beat to a corresponding segment in the Holter ECG recording to establish a synchronization point. The timestamp of other ICM-detected AF episodes was then adjusted to the timestamp of the Holter recording system based on the synchronization point.

### AI model

A convolutional neural network (CNN) model for classifying Assert-IQ-detected AF episodes was developed using manually adjudicated AF EGM signals randomly selected from US ICM devices that were remotely monitored on the Merlin.net patient care network for any reason of monitoring. The CNN model uses the device-stored EGM signal of each ICM-detected AF episode as the input and classifies each AF episode as true or false. The model is built upon a deep 1-dimensional CNN architecture designed for binary signal classification. It consists of sequential convolutional blocks, each comprising Conv1D layers followed by rectified linear unit activation, batch normalization, and dropout regularization. The number of filters and dropout rates increase progressively with depth to enhance feature learning and prevent overfitting. Max pooling layers are periodically incorporated to downsample feature maps and improve generalization. After the convolutional and pooling stages, a global average pooling layer condenses the learned features, which are then passed through fully connected dense layers. The final output is generated via a sigmoid activation function. The model is trained using the binary focal cross-entropy loss function and optimized with the Adam optimizer. None of the ICM-detected AF episodes in this study were used in the algorithm development process. The CNN model was applied to ICM-detected AF episodes in this study, and those AF episodes retained by the AI algorithm (ie, classified as true by the AI algorithm) were used to assess the AF detection performance of the Assert-IQ ICM system with the AI classification capability.

### Statistical analysis

ICM-detected AF episodes were compared with the gold standard, core laboratory–annotated AF events. The performance metrics, including sensitivity, specificity, positive predictive value (PPV), negative predictive value (NPV), and accuracy, were evaluated by comparing ICM-detected AF with that of simultaneous Holter monitoring. Uninterpretable ECG segments owing to noise or lead disconnection and ECG segments annotated as atrial tachycardia or atrial flutter were excluded from AF performance assessment.

**Figure 1:**
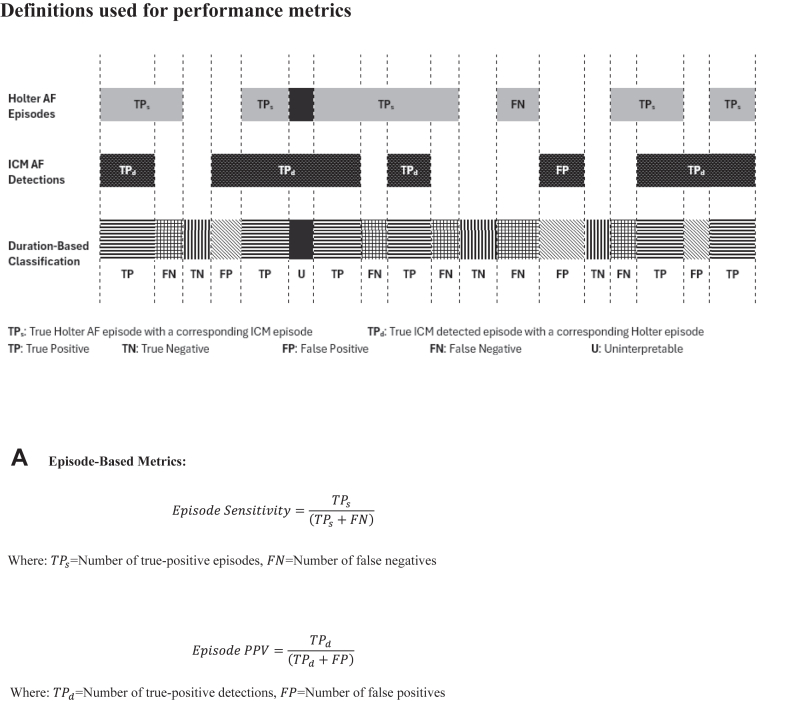

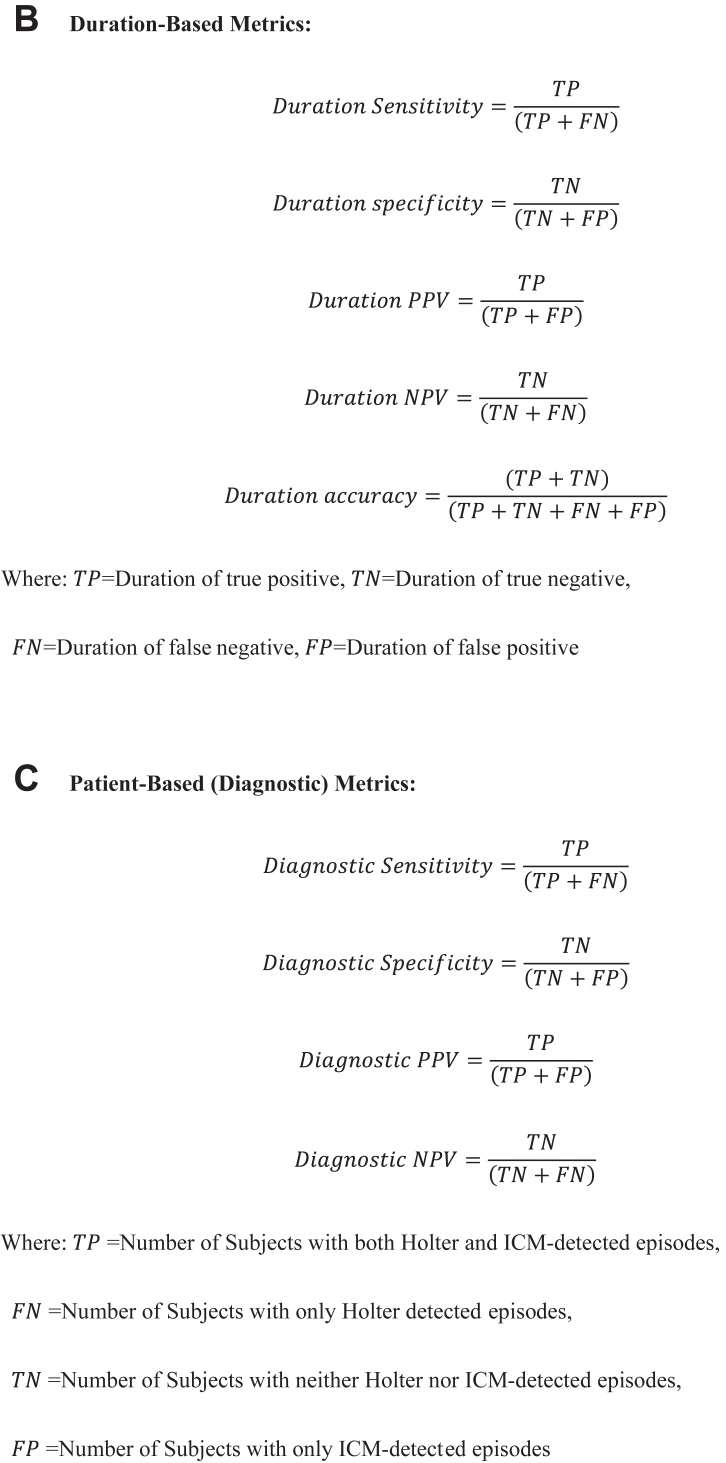
Formula for the calculation of episode-**(A)**, duration-**(B)**, and patient-based (diagnostic) **(C)** performance metrics. AF = atrial fibrillation; ICM = insertable cardiac monitor; NPV = negative predictive value; PPV = positive predictive value.

The performance characteristics of arrhythmia detection accuracy were evaluated using episode-, patient-, or duration-based approaches. How the performance metrics were calculated using the 3 approaches is presented in Figure 1. Episode-based metrics evaluate the proportion of true episodes and detected episodes that are correctly identified, irrespective of the patient who had the episode. Patient-based metrics, also called diagnostic metrics, evaluate the ability of the ICM to detect the presence or absence of AF within a specific patient, whereas duration-based metrics calculate the overlap of the duration of time between periods annotated as AF and periods detected as AF by the ICM, requiring that the ICM and the Holter are synchronized. The episode sensitivity and PPV were also estimated using the generalized estimating equation (GEE) estimates, which adjust for multiple episodes within a subject and account for the possible correlation of AF detection performance on episodes from the same Holter recording. Continuous variables were summarized by mean ± standard deviation. Categorical variables were summarized by frequency and percentage. Patient- and duration-based results are presented as percentages, whereas episode-based results are reported as percentages along with their 95% confidence intervals (CIs). The AF burden determined by the ICM was compared with the reference Holter AF burden by calculating the Pearson correlation coefficient between the paired measurements. Analyses were conducted using SAS 9.4.

## Results

### Study cohort

The study enrolled 151 subjects at 11 sites in the United States. Participants included 136 patients scheduled to receive an Assert-IQ ICM and 15 who were already implanted. The baseline characteristics of the enrolled subjects are presented in Table 1. The mean age of patients was 69.6 ± 10.0 years, and 61.6% were male. There was a history of hypertension in 76.2%, hyperlipidemia in 55.6%, stroke (including thromboembolism or transient ischemic attack) in 11.9%, paroxysmal AF in 79.5%, persistent AF in 19.2%, atrial flutter in 6.6%, and supraventricular tachycardia in 9.9%.

**Table 1:** Baseline demographics

Characteristic	Enrolled subjects (N = 151)
Age, mean ± SD	69.6 ± 10.0
Gender, % (n), female	38.4 (58)
BMI, mean ± SD	30.62 ± 6.96
Medical history, % (n)∗	
Diabetes	23.2 (35)
Hypertension	76.2 (115)
Hyperlipidemia	55.6 (84)
Stroke, TIA, or thromboembolism	11.9 (18)
Vascular disease	33.8 (51)
Congestive heart failure	25.8 (39)
NYHA class I	2.6 (4)
NYHA class II	7.9 (12)
NYHA class III	1.3 (2)
NYHA class IV	0.0 (0)
NYHA class unknown	13.9 (21)
LVEF†, mean ± SD	56.1 ± 8.7
LVEF of ≤30%	1.3% (2)
LVEF of 31%–40%	6.0% (9)
LVEF of >40%	90.1% (136)
Not reported	2.6% (4)

BMI = body mass index; LVEF = left ventricular ejection fraction; NYHA = New York Heart Association; SD = standard deviation; TIA = transient ischemic attack.

∗ Subject can be counted in more than 1 category/subcategory.

† With available data (147 subjects).

**Figure 2:**
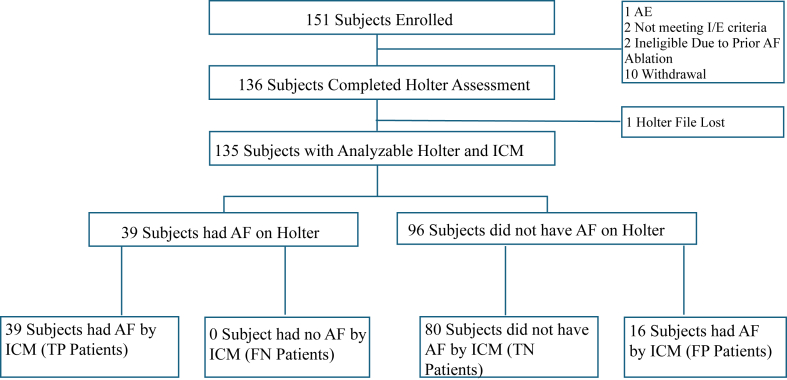
Study diagnosis. AE = adverse event; AF = atrial fibrillation; FN = false negative; FP = false positive; ICM = insertable cardiac monitor; I/E = inclusion/exclusion; TN = true negative; TP = true positive.

15 enrolled subjects were excluded from the final analysis: 2 subjects did not meet inclusion/exclusion criteria, 8 subjects withdrew prior to attempted ICM insertion, 2 subjects withdrew before Holter assessment, 1 subject required device removal owing to insertion site pain and red lines, and 2 subjects underwent AF ablation before Holter assessments. Therefore, a Holter assessment was performed in 136 patients. 1 additional patient’s Holter recording file was lost during transportation, resulting in 135 patients with complete analyzable Holter data (Figure 2).

### R-wave amplitude

The R-wave amplitude measured after insertion was 0.51 ± 0.38 mV (n = 130), with a 95% CI of 0.45–0.58 and a median of 0.45 (interquartile range 0.32–0.71). At the 1-month visit, the R wave was 0.49 ± 0.22 mV (n = 133), with a 95% CI of 0.45–0.52 and a median of 0.47 (interquartile range 0.29–0.64). There was no significant difference between the R wave at the insertion and the R wave at the 1-month visit (*P* = .43).

### Annotated AF events

A total of 19,283.2 hours of Holter recording time from 135 patients were analyzed. The average recording duration was 6.0 days per patient, with a median duration of 6.9 days. True AF was annotated in 39 of the 135 patients, resulting in a total of 522 true AF episodes lasting ≥2 minutes and 3939.8 hours of AF.

### Assert-IQ ICM AF detection performance

The ICM correctly identified all 39 patients with AF and 80 of 96 patients without AF, resulting in 100% sensitivity, 83.3% specificity, 70.9% PPV, and 100% NPV at the patient level. The episode- and duration-based performance metrics are presented in Table 2. In the episode-based results, gross sensitivity was 99.4% (95% CI 98.3–99.9), patient average sensitivity was 99.9% (95% CI 99.7–100), and GEE estimate sensitivity was 99.2% (95% CI 95.2–99.9), respectively. Gross PPV was 79.9% (95% CI 77.6–82.1), patient average PPV was 69.1% (95% CI 56.9–81.3), and GEE estimate PPV was 70.0% (95% CI 57.2–80.3), respectively. In the duration-based results, sensitivity was 93.0%, specificity was 99.3%, PPV was 97.4%, NPV was 98.0%, and the overall accuracy value was 97.9%. The AF burden detected by the Assert-IQ ICM vs annotated true AF burden showed a correlation coefficient of 0.99, with a mean bias of −0.9% (95% limits of agreement −13.6% to 11.6%) in a Bland-Altman analysis.

**Table 2:** ICM device performance analysis with AI integration

Performance metrics		All 135 patients at 1 mo	After AI classification
Patient-based results	Sensitivity (%)	100	100
	Specificity (%)	83.3	88.5
	Positive predictive value (%)	70.9	78.0
	Negative predictive value (%)	100	100
Episode-based results	Sensitivity (%) Gross (95% CI)	99.4 (98.3–99.9)	99.4 (98.3–99.9)
	Patient average (95% CI)	99.9% (99.7–100)	99.9% (99.7–100)
	GEE estimate (95% CI)	99.2% (95.2–99.9)	99.2% (95.2–99.9)
	Positive predictive value (%) Gross (95% CI)	79.9 (77.6–82.1)	93.6 (91.9–95.0)
	Patient average (95% CI)	69.1% (56.9–81.3)	76.5% (64.7–88.3)
	GEE estimate (95% CI)	70% (57.2–80.3)	86.1% (75.6–92.6)
Duration-based results	Sensitivity (%)	93.0	93.0
	Specificity (%)	99.3	99.5
	Positive predictive value (%)	97.4	98.1
	Negative predictive value (%)	98.0	98.0
	Accuracy value (%)	97.9	98.1

AI = artificial intelligence; CI = confidence interval; GEE = generalized estimating equation; ICM = insertable cardiac monitor.

### Impact of the AI algorithm on AF detection performance

The AI algorithm was applied to all ICM-detected AF episodes in the performance analysis. Among those 1256 episodes, 1004 were true detections and 252 were false detections compared with core laboratory–annotated AF events. The AI algorithm retained all 1004 true detections and 69 false detections, resulting in 100% sensitivity and a 72.6% false-positive reduction compared with Assert-IQ device detections.

The AI algorithm classification resulted in improvements in AF detection performance, as presented in Table 2. Sensitivity values remain unchanged because the AI algorithm did not falsely reject any true AF detection. PPV and specificity of AF detection were improved after the AI classification. In the patient-based results, specificity improved from 83.3% to 88.5% and PPV increased from 70.9% to 78.0%. In the episode-based results, gross PPV increased substantially from 79.9% (77.6%, 82.1%) to 93.6% (91.9%, 95.0%), patient average PPV rose from 69.1% (56.9%, 81.3%) to 76.5% (64.7%, 88.3 %), and GEE estimate PPV improved from 70.0% (57.2%, 80.3%) to 86.1% (75.6%, 92.6%). In the duration-based results, specificity improved from 99.3% to 99.5%, PPV increased from 97.4% to 98.1%, and accuracy improved from 97.9% to 98.1%.

The common reasons for Assert-IQ-detected false AF episodes were sinus arrhythmia (38.4%), frequent atrial and ventricular ectopy (31.3%), and noise artifact and oversensing (29.8%). The AI algorithm rejected false AF episodes in these categories by 81.4% (sinus arrhythmia), 62.9% (ectopy), and 74.0% (noise), respectively.

### PPV by device-detected episode duration

**Figure 3:**
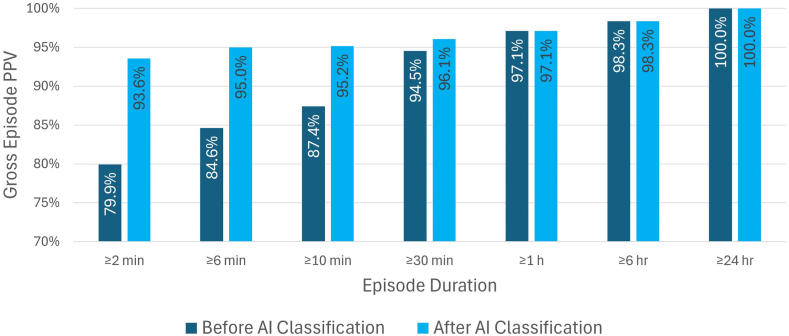
PPV of Assert-IQ ICM for AF detection before and after AI classification. PPV values are shown as gross (episode-based) calculations across various AF episode duration thresholds. AI implementation significantly improved detection accuracy, particularly for shorter AF episodes. AF = atrial fibrillation; AI = artificial intelligence; ICM = insertable cardiac monitor; PPV = positive predictive value.

Gross PPV changes by various device-detected episode duration cutoffs and the AI algorithm are presented in Figure 3. Among device-detected AF episodes, PPV progressively improved with increasing episode duration requirements (eg, 79.9%, 87.4%, and 97.1% for episodes lasting ≥2 minutes, ≥10 minutes, and ≥1 hour, respectively) and achieved 100% for episodes lasting ≥24 hours. The AI algorithm improved PPV values, particularly when shorter-duration episodes were included in the assessment.

## Discussion

This study evaluated AF detection performance of Assert-IQ ICMs by comparing device-detected episodes with the ground truth determined by a core laboratory using continuous Holter ECG assessment. The main findings of this study are that (1) Assert-IQ ICM is highly sensitive in detecting AF events and accurately quantifies AF burden; (2) PPV of ICM detections progressively increase with longer episode duration requirement, achieving 97.1% for episodes lasting ≥1 hour and 100% for ≥24 hours; and (3) the AI algorithm was highly effective in eliminating false-positive AF detections without any loss in sensitivity, thus further improving PPV and specificity while maintaining sensitivity.

These findings demonstrate significant improvements in AF detection performance compared with earlier generations of ICMs by Abbott. The DETECT-AF study, which evaluated the Confirm Rx DM2102 ICM AF detection performance using the same methodology, reported lower performance results (eg, episode-based gross sensitivity of 94.5% and PPV of 64.0%, duration-based sensitivity of 83.9%) for episodes lasting ≥2 minutes.^7^ In addition, the Assert-IQ ICM showed higher PPV values at all duration thresholds. These comparisons highlight the effectiveness of the enhanced AF detection algorithm in the Assert-IQ device firmware for high detection accuracy and fewer false-positive detections, addressing a key limitation identified in previous ICM generations.

Despite the advancements in ICM device firmware, the accuracy of AF detection can be further improved by more sophisticated algorithms beyond the device level. AI and, more specifically, neural network models have recently emerged as powerful tools in various health care applications, including classifying EGMs recorded by ICMs.^10^, ^11^, ^12^^,^^14^ To the best of our knowledge, this is the first study reporting the application of a CNN algorithm to Assert-IQ-detected AF episodes. The classification results by this AI algorithm are encouraging. It retained all true-positive episodes and rejected 72.6% false-positive episodes triggered by Assert-IQ devices, resulting in a significant improvement in episode-based PPV values and particularly among AF episodes with shorter duration. Short-duration AF episodes are common and can be challenging to adjudicate owing to their transient nature and potential for artifact. The observed improvement in PPV with AI support, especially in these shorter episodes, suggests a meaningful role for AI in reducing false positives and supporting more efficient clinical workflows. This AI algorithm is also effective in eliminating false AF episodes owing to different reasons, such as sinus arrhythmia, frequent atrial and ventricular ectopy, and noise. Implementing this AI algorithm on the remote monitoring server may alleviate the clinical burden associated with ICM data review and reduce the clinical cost and time required for arrhythmia diagnosis and management. The AI algorithm received recent regulatory approval from both the US Food and Drug Administration and the Japan Pharmaceuticals and Medical Devices Agency and has since been commercially launched in the United States.

### Clinical implications

The perfect sensitivity and NPV at the patient level have significant clinical implications. In AF management, missing episodes, especially in patients being evaluated for anticoagulation, can lead to undertreatment. The device correctly identified every patient with AF in our cohort.

The progressive improvement in PPV with increasing AF duration thresholds suggests that the Assert-IQ ICM achieves optimal performance for longer episodes, which are clinically more significant for treatment decisions.^15^^,^^16^ Although the 2-minute threshold ensures the detection of even brief AF episodes, it also increases false positives, potentially leading to clinical uncertainty. The longer AF detection thresholds (eg, ≥6 minutes) offer enhanced diagnostic confidence, which is consistent with evidence that long device-detected AF (at least 6 minutes) is associated with an increased risk of ischemic stroke or systemic embolism.^15^

Accurate quantification of AF burden is increasingly important. Subclinical AF, detected only by continuous monitoring devices, is a known thromboembolic risk factor.^17^ The LOOP study found that ICMs can detect subclinical AF in approximately 30% of individuals older than 70 years with cardiovascular risk factors.^18^ The ARTESiA trial (NCT01938248) demonstrated that apixaban reduced the risk of stroke in patients with device-detected subclinical AF, albeit with an increased risk of major bleeding.^19^ A recent meta-analysis combining ARTESiA and NOAH-AFNET 6 further supports the potential benefit of direct oral anticoagulants in this population.^20^

### Limitations

Our study had several limitations. First, the detection performance was evaluated against the Holter assessment for up to 7 days. Although this assessment window was longer than previous studies of a similar nature, so that the results are more representative of ICM performance than those assessments limited to just 1 or 2 days, the long-term detection performance over the ICM device lifespan could not be fully evaluated in this type of study. Second, the enrolled subjects were known patients with AF. As a result, AF prevalence in the study was higher than that in the general ICM population or patient subgroups such as syncope and cryptogenic stroke. Given that AF detection PPV partially depends on AF prevalence, the performance may vary and decrease in real-world experience. However, the reported sensitivity and specificity values for AF detection remain true because they are not affected by disease prevalence. Third, we focused specifically on AF episodes of 2 minutes or greater in duration, so our results do not apply to episodes of shorter duration. In addition, although Holter monitoring serves as our gold standard, it is not perfect either. Signal quality issues and interpretation variations could potentially affect the performance assessment. Finally, although the AI algorithm exhibited strong performance in this study, advancing the interpretability and explainability of AI systems continues to be a priority for future research.

## Conclusion

The Assert-IQ ICM demonstrates excellent performance in detecting AF and quantifying AF burden. The AI-based episode classification algorithm, which is not yet commercially available, shows promise for further improving the specificity and PPV of AF detection. These findings suggest that AI integration represents a significant advancement in ICM technology, maintaining excellent sensitivity while substantially reducing false positives, thereby enabling more efficient and accurate AF management.

## Disclosures

U.B.: Consultant for Abbott, MDT, BSC, Biotronik, and Philips. G.M.: Consultant for Abbott, Boston Scientific, and Medtronic. M.J.: Consultant for Abbott, Medtronic, Biosense Webster/Johnson & Johnson MedTech EP, and Philips. F.C.: Consultant for Boston Scientific and Abbott and equity/advisory board for CyndRx. M.K.: Consultant for Abbott. R.G.: Consultant for Abbott, Johnson & Johnson, Boston Scientific, and Sanofi. D.Y.: Consultant for Abbott. L.F., F.Q., W.L., K.L., and V.C.: Employees of Abbott. S.M.: Consultant for Abbott, Boston Scientific, and Medtronic. D.L.: Consultant for Abbott, AtriCure, Medtronic, Boston Scientific, and Johnson & Johnson MedTech. No other authors have conflicts of interest to disclose.
